# The Relationship between Vitamin D and Periodontal Pathology

**DOI:** 10.3390/medicina54030045

**Published:** 2018-06-12

**Authors:** Eglė Jagelavičienė, Inga Vaitkevičienė, Dovilė Šilingaitė, Eglė Šinkūnaitė, Goda Daugėlaitė

**Affiliations:** 1 Department of Dental and Oral Pathology, Medical Academy, Lithuanian University of Health Sciences, Eivenių 2, Kaunas LT-50161, Lithuania; vaitingai@gmail.com (I.V.); sinkunaite.egle@gmail.com (E.Š.); 2 AND Klinika, Žirmūnų 107, Vilnius LT-09116, Lithuania; dovile.silingaite@gmail.com; 3 Faculty of Medicine, Medical Academy, Lithuanian University of Health Sciences, A. Mickevičiaus 9, Kaunas LT-44307, Lithuania; Goda.daugelaite@gmail.com

**Keywords:** osteoporosis, jaw, vitamin D deficiency, bone mineral density (BMD), 25-hydroxyvitamin D, serum levels

## Abstract

Osteoporosis and periodontal diseases are common problems among the elderly population. Vitamin D is a secosteroid hormone that is either synthesized by human skin cells under the effect of UV radiation or consumed through diet. Deficiency in vitamin D leads to reduced bone mineral density, osteoporosis, the progression of periodontal diseases and causes resorption to occur in the jawbone. Sufficient intake of vitamin D can decrease the risk of gingivitis and chronic periodontitis, as it has been shown to have immunomodulatory, anti-inflammatory, antiproliferative effects and initiates cell apoptosis. In addition, vitamin D is also important for bone metabolism, alveolar bone resorption and preventing tooth loss. It increases antibacterial defense of gingival epithelial cells and decrease gingival inflammation, improves postoperative wound healing after periodontal surgery and is an important supplement used as prophylaxis in periodontology. This publication aims to update the recent advances, stress the clinical importance, and evaluate vitamin D in the prevention of periodontal diseases to reach a successful outcome of conservative and surgical treatment. An analysis of the literature shows that vitamin D plays a significant role in maintaining healthy periodontal and jaw bone tissues, alleviating inflammation processes, stimulating post-operative healing of periodontal tissues and the recovery of clinical parameters. However, further research is needed to clarify the required vitamin D concentration in plasma before starting periodontal treatment to achieve the best outcome.

## 1. Introduction

Vitamin D is a secosteroid, which is synthesized from 7-dehydrocholesterol during a photochemical reaction under the effect of ultraviolet radiation on the skin or is consumed through digestion [1]. Vitamin D_3_ is further hydroxylated in the liver into 25-hydroxyvitamin D_3_ (25(OH)D_3_) [2]. This is the main and most stable form of vitamin D in blood plasma. It is a biologically active metabolite, one of the functions of which is maintaining the balance of calcium and phosphorus concentration in blood by regulating their absorption in the intestines and reabsorption in kidney. It also assists in promoting the remodeling of the bones [2,3]. Constant low uptake of vitamin D and calcium leads to a negative calcium balance, disrupted bone mineralization, and loss of bone structure [1]. Vitamin D deficiency leads to rickets in children, and osteoporosis (OP) in adults as well as an increased probability of bone fracture [1,4]. 1,25(OH)_2_D_3_ is vital to the immune system as it stimulates the non-specific immune response to fight against infectious diseases [2,5]. Vitamin D receptors (VDR) in monocytes, macrophages, neutrophils and dendritic cells bind to 1,25(OH)_2_D_3_ molecules and stimulates the release of antimicrobial peptides [5]. Such regulation of the immune system is an important defense mechanism for digestive, respiratory and genitourinary system cells, skin, eyes, and mouth [6]. Vitamin D affects the pathogenesis of periodontal diseases (PD) via immunomodulation, increases bone mineral density (BMD), reduces bone resorption, and is important in fighting against agents that cause periodontal diseases. There has been an increase in interest in and publication of articles investigating the importance of vitamin D in prophylaxis and treatment of dental caries and periodontal diseases [6]. The purpose of the following literature analysis is to analyze the role of vitamin D in the pathogenesis of PD, its influence on the quality of the alveolar bone, the correlation between 25-hydroxyvitamin D concentration in plasma and PD as well as the importance of vitamin D in chronic periodontitis prophylaxis and treatment.

## 2. Significance of Vitamin D in Immune Response of Periodontium

Liu et al. discovered that, during an inflammatory process, dental pulp fibroblasts and periodontal cells produce 25-hydroxylase, which stimulates the production of 25(OH)D_3_ [7]. As pathological microorganisms affect the cell membrane receptors, 1α-hydroxylase synthesis is activated, during which 1,25(OH)D_3_ is formed from 25(OH)D_3_ [6]. The resulting molecule binds with the VDR in the immune and epithelial cells and participates in the epithelium defense mechanism against the pathogen [5,6]. 1,25(OH)D_3_ activates the synthesis of proteins which are required in the tight, gap and desmosome junctions of epithelial cells [6]. The junctional epithelium connects to the tooth through loose junctions, thus, creating favorable conditions for a bacterial invasion from dental plaque, which initially causes inflammation of the periodontal tissue (PT), and, as the process advances, resorption and tooth loss occurs [8].

1,25(OH)D_3_ regulates the non-specific immune response, activates hydrogen peroxide secretion in monocytes, stimulates the synthesis of antimicrobial peptides, e.g., β-defensin and cathelicidin LL-37. Cathelicidin LL-37 plays a role in chemotaxis, production of cytokines and chemokines, cellular reproduction, vascular permeability, wound healing, and neutralization of bacterial endotoxins [6]. McMahon et at. studied human gingival cell cultures and the effect of vitamin D on the expression of non-specific immune system of these cells. After affecting gingival cell cultures with 1,25(OH)D_3_, cathelicidin, LL-37 secretion increased, and its antimicrobial effect against *Actinobacillus actinomycetemcomitans* lasted for 24 h [9].

1,25(OH)D_3_ takes place in specific immune system by affecting B-lymphocytes and T-lymphocytes [6]. These cells emit cytokines and immunoglobulins, and they specifically destroy bacterial pathogens which are transferred by macrophages and dendritic cells. Such immune processes harm the PT and aggravate the course of PD. Vitamin D suppresses the proliferation of T-lymphocytes, secretion of immunoglobulins, transformation of B-lymphocytes into plasma cells, it inhibits the unwanted process, and protects the organism from excessive specific immune response by decreasing the secretion of IL-1, IL-6, IL-8, IL-12, TNFα cytokines [1,7]. These cytokines are released in PD pathogenesis during a bacterial invasion. They cause lymphocyte infiltration, bone resorption, deterioration of extracellular matrix. Tang et al. studied the human periodontal tissue cell cultures trying to discern the anti-inflammatory effect of vitamin D on the cells. Less IL-8 was discovered in the cell cultures affected by *Porphyromonas gingivalis* and 1,25(OH)D_3_ than in cell cultures affected only by *Porphyromonas gingivalis*. Thus, the hypothesis that vitamin D is effective in PD prophylaxis and treatment was confirmed [10]. Teles et al. confirmed anti-inflammatory properties of vitamin D by determining that higher concentrations of vitamin D in blood serum contain less IL-6 and leptin and more adiponectin, which regulates the immune response. An increase in leptin signifies the presence of an infectious process and inflammation (proliferation and activation of T-lymphocytes, production of cytokines), adiponectin suppresses the production and activity of cytokines [11].

## 3. Concentration of 25-Hydroxyvitamin D in Plasma and Periodontal Disease

The quantity of vitamin D in a human organism is determined by the concentration of its metabolite 25(OH)D_3_ in plasma; it normally fluctuates from 25 to 138 nmol/L. A concentration lower than 37.5 nmol/L shows vitamin D deficiency. A concentration higher than 200 nmol/L signifies hypervitaminosis [12]. To have an effect on periodontium, the plasma concentration should reach 90–100 nmol/L [3]. The correlation between varying 25(OH)D_3_ plasma concentrations and PD is shown in Table 1.

During acute periodontal inflammation, 25(OH)D_3_ concentration increases due to increased 25-hydroxylase activity of periodontal cells. During a chronic inflammation, it decreases [19]. Due to the production of this enzyme in aggressive periodontitis, 25(OH)D_3_ concentration in periodontal pockets is 300 times greater than in blood plasma [7]. Zhang et al. argue that, in the case of aggressive periodontitis, an increase in IL-6 and 25(OH)D_3_ concentration, leukocytes and neutrophils is observed [20]. Low concentration of 25(OH)D_3_ in blood plasma indicates vitamin D deficiency, unbalanced immune reactions in the organism, and the progression of periodontal disease [18].

## 4. Significance of Vitamin D in Mandibular Bone

The effect of vitamin D and calcium supplements in treating systemic BMD decrease is based on its activity—regulation of calcium and phosphorus concentration in blood [2,3]. Constant low ingestion of vitamin D and calcium leads to a negative calcium balance, disrupted bone mineralization, and loss of bone structure [1]. Vitamin D deficiency causes rickets in children and OP in adults [1] as well as an increased risk of bone fracture [1,5]. The optimal 25(OH)D_3_ recommended concentration in blood plasma for skeletal bone tissue is no lower than 80 nmol/L, for periodontal tissue—approximately 90–100 nmol/L. Lower concentrations are associated with periodontal disease progression and tooth loss [3].

Mandibular bone is one of the four tissues forming the periodontium. For this reason, OP and periodontal disease are closely related. In case of OP, the BMD decreases in all bones, including the jawbone, where the resorption of the alveolar ridge and tooth loss increases [2,21]. After studying 400 older women in 2010, Al Habashneh et al. determined that women with lower skeletal BMD display a more progressed form of chronic periodontitis. They were also diagnosed with increased alveolar process resorption than with normal BMD. A survey of these participants revealed that women who took vitamin D suffered from chronic periodontitis less often than those who did not [22].

The systemic increase of cytokines, which influence bone resorption in the entire skeleton and jawbone, can be noted in people with low systemic BMD. As mentioned previously, periodontal infection causes a local increase in cytokines which stimulates the activity of osteoclasts and bone resorption [23]. In 2011, Jabbar et al., studied the correlation between cytokine and 25-hydroxyvitamin D concentration in blood plasma in OP. The study subjects were 185 women of postmenopausal age suffering from OP and 185 healthy women of the same age. The study showed that 25-hydroxyvitamin D concentration in blood plasma of the subjects was significantly lower than that of the control group (66.62 nmol/L and 97.21 nmol/L, respectively), while the quantity of cytokines, receptor activator of nuclear factor κB ligand (RANKL) and osteoprotegerin (OPG) were significantly higher. The RANKL and OPG balance is regulated by glucocorticoids, vitamin D, and oestrogens [24]. Wactawski-Wende et al. argued that due to vitamin D’s ability to increase BMD and reduce resorption, vitamin D_3_ supplements may be used in prophylaxis and treatment of periodontal disease in postmenopausal age women [23].

## 5. Vitamin D in Prophylaxis and Treatment of Periodontal Diseases

In case of vitamin D hypovitaminosis, getting less than 400 IU of vitamin D daily may cause a decrease of calcium concentration in blood plasma, an increase of parathyroid hormone secretion, disruption of bone tissue remodeling, and may develop a secondary hyperparathyroidism [25]. The recommended daily dosage of vitamin D for people under 70 years old is 600 IU; for people older than 70 years—800 IU [26]. During vitamin D deficiency treatment, daily dosage may be increased to 2000 IU, without tracking 25(OH)D_3_ concentration in blood plasma. If the daily dosage of vitamin D reaches 40,000 IU, vitamin D hypervitaminosis may occur in healthy individuals. This quantity of vitamin D taken daily for extended periods of time disrupts calcium metabolism, causes parathyroid gland hyperfunction [3], and forms conditions for the development of nephrolithiasis [27]. Approximately only 90 IU of vitamin D may be absorbed from food every day without consumption of supplements. Human body synthesizes approximately 10,000 IU of vitamin D from tanning under natural sunlight until light redness of the skin. The recommended dosage of vitamin D may be absorbed by exposing the face, hands, and palms to natural sunlight 2–3 times a week without reaching light redness of the skin [3]. Hildebolt investigated the correlation between the daily intake of vitamin D supplements and the increase in 25(OH)D_3_ concentration in blood plasma. According to Hildebolt, consuming 200 IU of vitamin D supplements per day increases the concentration of 25(OH)D_3_ in blood plasma by 10 nmol/L, whereas consuming 1000–2000 IU increases it by 47 nmol/L [3].

Bashutski et al. published the results of a long-term clinical study where the correlation between the quantity of vitamin D in blood plasma and periodontal surgeries was studied. Researchers determined that research subjects with a vitamin D deficiency in blood plasma showed less effective results (lower tissue attachment level and probing depth change) after periodontal surgery. The authors argue that to improve post-surgery results, it is advised to examine vitamin D level in the patients’ blood prior to the treatment and avoid vitamin D deficiency by taking supplements [27].

Alshouibi et al. studied the correlation between the quantity of vitamin D and state of periodontium in 562 older men. Study results showed that subjects who received more than 800 IU of vitamin D daily had a lower risk of having a more severe form of chronic periodontitis (results were based on probing depth, attachment level, loss of alveolar bone), whereas those receiving less than 400 IU of vitamin D suffered from a more advanced level of alveolar bone resorption [28].

Hiremath et al. conducted a random sampling of clinical research to prove the anti-inflammatory effect of vitamin D on gingiva. Results showed that a 500–2000 IU dosage of vitamin D is safe and effective in gingival inflammation treatment. Notable results were shown after three months. Results were observed after one month where subjects took a dose of 2000 IU of vitamin D or higher. Consumption of higher doses led to a change in 25(OH)D_3_ concentration in blood plasma. While not consuming vitamin D, the concentration increased by 0.87 nmol/L. During consumption of 500 IU, the concentration increased by 32.03 nmol/L. During consumption of 1000 IU, the concentration increased by 42.12 nmol/L. During consumption of 2000 IU, the concentration increased by 74.22 nmol/L [29]. This randomized clinical trial demonstrates the effect of vitamin D on gingival inflammation and the required vitamin D dosage to reach this effect.

## 6. Conclusions

In conclusion, vitamin D is significant in periodontology as it takes part in the synthesis of proteins that are needed in formation of mucous membrane. This creates a physical barrier and, thus, hinders the transfer of pathogens further into deeper tissues. The antimicrobial protein synthesis by immune and epithelial cells as well as non-specific immune responses are activated. Vitamin D also takes part in specific immune response by suppressing the destructive effect of chronic periodontitis. Moreover, it maintains systemic and jawbone density homeostasis. In order to prescribe vitamin D for treatment of periodontal diseases, further research is needed.

## Figures and Tables

**Table 1:** The relationship between 25-hydroxyvitamin D_3_ (25(OH)D_3_) concentrations in the plasma and periodontal diseases.

Authors, Year of Publication	Study Design	Sample Size	Outcome Measure	Outcome Measurement	Results
Dietrich et al., 2004 [13]	cross-sectional	11 202	Periodontitis	attachment level	decreased concentration is associated with changed (poor) periodontal condition
Dietrich et al., 2005 [14]	cross-sectional	6 700	Gingivitis	level of gingival inflammation (bleeding index)	decreased concentration is associated with gingival inflammation and higher bleeding index
Borggess et al., 2011 [15]	case-control	123 cases, 123 controls	PD in pregnant women	probing depth, bleeding index	women with vitamin D deficiency in the plasma (<75 nmol/L) are more prone to chronic periodontitis during pregnancy
Zhou et al., 2012 [16]	case-control	193 cases, 181 controls	PD and chronic obstructive pneumonia	pockets depth, periodontal attachment level, gingival bleeding index, teeth number	decreased concentration is associated with poor periodontal condition
Teles et al., 2012 [11]	exploratory	56	Chronic periodontitis	bleeding index, probing depth, periodontal attachment level, teeth number	decreased concentration is associated with poor periodontal condition
Antonoglou et al., 2013 [17]	comprehensive	80	Chronic periodontitis with type 1 diabetes	amount of plaque, probing depth, attachment level	authors did not find correlation between 25(OH)D3 concentration in the plasma and chronic periodontitis
Millen et al., 2013 [18]	multi-center	920	Chronic periodontitis in postmenopausal age	X-ray, attachment level, probing depth, bleeding index	decreased concentration is associated with chronic periodontitis
					increased concentration is associated with gingival bleeding
Liu et al., 2009 [19]	preliminary	178	Aggressive periodontitis	probing depth, attachment level, bleeding index	increased concentration is associated with aggressive periodontitis
Zhang et al., 2013 [20]	case-control	44 cases, 32 controls	Generalized aggressive periodontitis	probing depth, attachment level, bleeding index	increased concentration is associated with generalized aggressive periodontitis
